# Invasive neurophysiology and whole brain connectomics for neural decoding in patients with brain implants

**DOI:** 10.21203/rs.3.rs-3212709/v1

**Published:** 2023-09-20

**Authors:** Timon Merk, Richard Köhler, Victoria Peterson, Laura Lyra, Jonathan Vanhoecke, Meera Chikermane, Thomas Binns, Ningfei Li, Ashley Walton, Alan Bush, Nathan Sisterson, Johannes Busch, Roxanne Lofredi, Jeroen Habets, Julius Huebl, Guanyu Zhu, Zixiao Yin, Baotian Zhao, Angela Merkl, Malek Bajbouj, Patricia Krause, Katharina Faust, Gerd-Helge Schneider, Andreas Horn, Jianguo Zhang, Andrea Kühn, R. Mark Richardson, Wolf-Julian Neumann

**Affiliations:** Charité Universitätsmedizin Berlin; Charité Universitätsmedizin Berlin; Instituto de Matemática Aplicada del Litoral IMAL; Charité Universitätsmedizin Berlin; Charité Universitätsmedizin Berlin; Charité Universitätsmedizin Berlin; Charité Universitätsmedizin Berlin; Charité Universitätsmedizin Berlin; Massachusetts General Hospital; Massachusetts General Hospital; Massachusetts General Hospital; Charité Universitätsmedizin Berlin; Charité Universitätsmedizin Berlin; Charité Universitätsmedizin Berlin; Charité Universitätsmedizin Berlin; Beijing Tiantan Hospital; Beijing Tiantan Hospital; Beijing Tiantan Hospital; Medical School Berlin (MSB); Charité Universitätsmedizin Berlin; Charité Universitätsmedizin Berlin; Charité Universitätsmedizin Berlin; Charité Universitätsmedizin Berlin; Charite University; Beijing Tiantan Hospital; Charité - Universitätsmedizin Berlin; University of Pittsburgh; Charité Universitätsmedizin Berlin

## Abstract

Brain computer interfaces (BCI) provide unprecedented spatiotemporal precision that will enable significant expansion in how numerous brain disorders are treated. Decoding dynamic patient states from brain signals with machine learning is required to leverage this precision, but a standardized framework for identifying and advancing novel clinical BCI approaches does not exist. Here, we developed a platform that integrates brain signal decoding with connectomics and demonstrate its utility across 123 hours of invasively recorded brain data from 73 neurosurgical patients treated for movement disorders, depression and epilepsy. First, we introduce connectomics-informed movement decoders that generalize across cohorts with Parkinson’s disease and epilepsy from the US, Europe and China. Next, we reveal network targets for emotion decoding in left prefrontal and cingulate circuits in DBS patients with major depression. Finally, we showcase opportunities to improve seizure detection in responsive neurostimulation for epilepsy. Our platform provides rapid, high-accuracy decoding for precision medicine approaches that can dynamically adapt neuromodulation therapies in response to the individual needs of patients.

The advent of clinical brain computer interfaces (BCI) that can adapt neurostimulation therapy to therapeutic demand signifies a unique moment in medical history. Closed-loop therapies have begun to automatize therapeutic adjustment based on signals recorded directly from the brain and body of patients^1–5^, with millisecond precision. Raw signals from intracranial electroencephalography (iEEG) electrodes are increasingly available to be transformed into input features for developing machine learning-based therapeutic control policies that can adapt therapy in real-time. Such strategies have unprecedented potential to improve the quality of lives of millions of patients with brain disorders, by reacting precisely and continuously to fluctuating signals of patient-specific brain states.

To realize the full potential of BCIs, robust evaluation and implementation of machine learning models must be enabled. Three major obstacles, however, stand in the way: 1) Machine learning can capitalize on large datasets but current brain signal decoding approaches for BCI still commonly rely on individual patient training sets of short duration. This reliance is impractical for broad clinical adoption and significantly limits the utility of complex models for generalizable performance. 2) Current advances in basic neurosciences have revealed the presence of complex brain signal features such as waveform shape and aperiodic activity^6,7^, but standardized methods to extract them for machine learning applications are lacking. 3) Human behavior is encoded in whole brain circuit interactions, but current brain signal decoding solutions often fail to account for the underlying brain networks that give rise to the recorded signals^8^. To overcome these roadblocks, we developed a modularized open-source software platform for invasive brain signal decoding in neuromodulation via BCIs.

In this paper, we introduce this methodology and highlight its utility in a large cohort of patients across thousands of recording sources from invasive brain implants within three key areas of clinical neurotechnology innovation: movement disorders, psychiatry, and epilepsy.

## Results

### Processing of invasive neurophysiology data for machine learning based brain signal decoding

**Figure 1:**
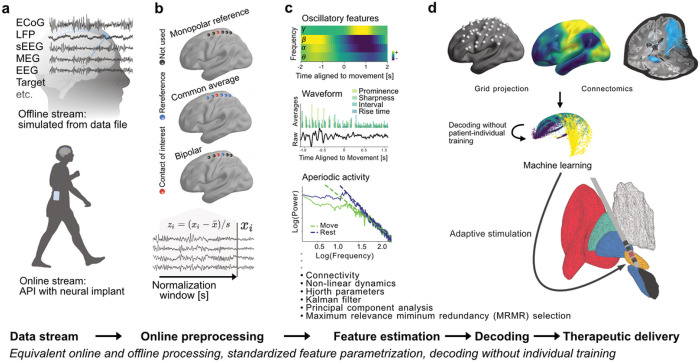
Use of brain signal decoding and adaptation of neurostimulation implemented in the py_neuromodulation platform. Neural data streams (a) can either be simulated from stored offline storage or streamed in real-time through direct connection to neural implants. Preprocessing (b) includes re-referencing, notch-filtering, downsampling, normalization, artifact detection and more, and was optimized for causal and computational efficient application. Multiple brain signal feature modalities (c) can be extracted that are relevant for invasive decoding: oscillatory activity, temporal waveform shape, oscillatory bursts, nonlinear dynamics, periodic and aperiodic power spectral components and more (Supplementary Table 1). Features can be mapped in space (d) for patient individual or across-patient decoding and consecutive adjustment of therapeutic delivery. Cross-validation, model evaluation metrics, and model architectures can be specified through the scikit-learn^9^ or alternative machine learning frameworks.

We developed an open, integrative and modularized platform (Fig. 1), for standardized and reproducible implementation of machine learning based brain signal decoding algorithms (https://github.com/neuromodulation/py_neuromodulation).

The modularized feature estimation chains include a versatile set of state-of-the-art signal processing algorithms. In brief, oscillatory dynamics, waveform shape, interregional coherence^6,7,10,11^ and more can be extracted through the modular architecture which further allows for flexible extension for more advanced feature estimation metrics, such as direction of information flow with granger causality or phase amplitude coupling^12^ (Supplementary Table 1). A key problem for invasive brain signal decoding is the individualized localization of brain implants across patients, which significantly complicates the development of large-scale models that generalize across patients. To address this, we developed dedicated approaches for across-patient decoding based on normative MRI connectomics and latent embeddings from contrastive learning with CEBRA^13^. In the following, we highlight the utility of this platform across three invasive brain stimulation use-cases.

#### Generalizable models for movement decoding across 1480 invasive recordings with prospective real-time validation

**Figure 2:**
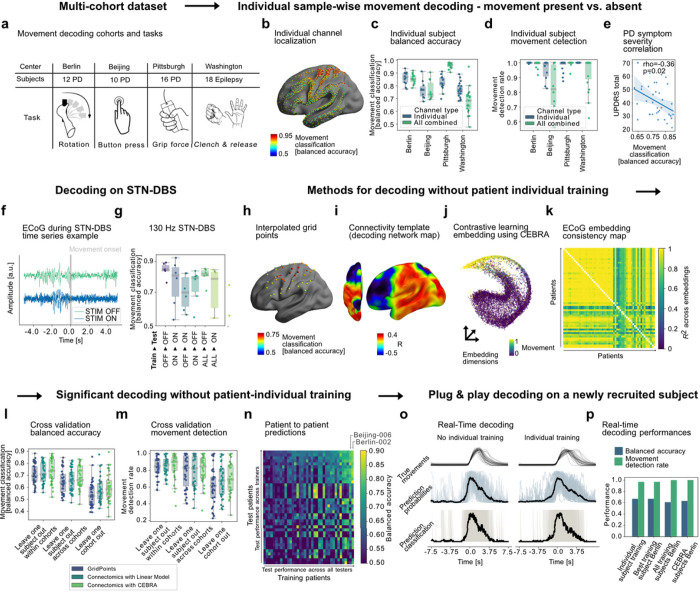
Movement decoding across patients, cohorts, diseases, movement types, and stimulation conditions. (a)Data from four cohorts with different diseases and movement types were used for decoding (1480 channels, 56 patients). (b) Individual recording locations with color-coded movement decoding classification performances. (c) Performances from patient individual 3-fold cross-validation. (d) Movement detection rates are defined as 300 ms consecutively correct classification during movement (98 ± 4 % for best channels across all patients). (e) In PD, mean channel performances negatively correlated with motor sign severity (UPDRS-III). (f) Exemplar time-series with DBS on ECoG raw data in a representative subject from the Berlin cohort. (g) Sample-wise performances OFF and ON clinically effective subthalamic 130 Hz DBS in six PD patients from Berlin (all above chance). To demonstrate the utility of *py_neuromodulation* for across-patient decoding, three alternative pipelines integrate channel selection and neural signals: (h) Spatial interpolation to a common grid in MNI space; (i) channel selection based on normative fMRI connectivity correlation to a predefined optimal decoding network; (j) embedding (exemplar subject shown) using contrastive learning with a convolutional neural network using CEBRA^13^. (k) shows embedding consistency from each to every other patient via linear identifiability. All three methods achieved high decoding accuracies within and across cohorts for sample-wise balanced accuracy (l) and movement detection rates (m). Patient to patient training (n) revealed interindividual variability for training other subjects vs. being trained on other subjects (n is ordered according to performance, subjects Berlin-002 and Beijing-005 were the best trainers). To demonstrate the ability to decode movements without patient individual training, we prospectively recruited one subject in Berlin and decoded movements using pretrained models based on all previous subjects of the Berlin cohort (o). Real-time decoding performances (p) show that *py_neuromodulation* can facilitate prospective real-time decoding based on various training cohorts without requiring individual training with high above chance sample-wise balanced accuracy and movement detection rates.

For the treatment of movement disorders, the decoding of volitional and pathological motor output has the potential to provide critical information for therapeutic adjustment for different diseases such as Parkinson’s disease or essential tremor^1,4^. We implemented *py_neuromodulation* for electrocorticography (ECoG) based movement decoding without individual training (56 patients, 1480 channels, mean age: 50.6 ± 17.8, 24 females) from three independent PD (n=38; Berlin/Pittsburgh/Beijing) and one epilepsy cohort (n=18 from Washington^14^) performing different upper limb movements (Fig. 2a, Supplementary Tables 2 and 3). In PD patients, ECoG strips were introduced through DBS burr-holes^15^. In a sub-cohort of six patients (Berlin), recordings were repeated during clinically effective STN-DBS.

First, patient specific movement classification performances based on ECoG signals were analyzed. We trained ridge regularized logistic regression classifiers and evaluated their performance using the balanced accuracy metric with 3-fold cross-validation on consecutive data segments, at the single sample level (100 ms precision indicating presence or absence of movement) and at the individual movement level (300 ms or more movement time decoded consecutively). Performance was significantly above chance in every subject, with an average balanced accuracy of 0.8/0.98 ± 0.07/0.04 for single sample/movement detection in the best channel per subject (Fig. 2b–d).

Importantly, we could identify two key factors associated with relatively lower performances that will be crucial to account for in future clinical applications: disease severity and therapeutic DBS. First, we show that decoding performance is negatively correlated with clinical symptom severity in PD patients, as measured with Unified Parkinson’s Disease Rating Scale (Spearman’s rho = −0.36; p = 0.02) (Fig. 2e). We reported a similar result earlier for the Pittsburgh cohort^11^, and therefore repeated the analysis for Berlin and Beijing alone, excluding all previously reported data, and could empirically reproduce the negative correlation across new cohorts (rho=−0.43, p=0.03). We may speculate that neurodegeneration in PD may impact neural encoding of movement, which may also impede machine learning based decoding performance. Next and equally important, we show that therapeutic electrical stimulation (130 Hz STN-DBS) can significantly deteriorate sample-wise decoding in some but not all patients and that models trained separately for OFF and ON stimulation conditions outperform models trained on either condition alone. Nevertheless, movement detection remained acceptable with 0.88 ± 0.17 movement detection rate across movements even during high-frequency DBS (Fig. 2f,g). Our results highlight the necessity to account for therapies and disease related brain state changes in clinical BCI.

The more serious limitation of the abovementioned results is the dependence on patient individual training. For a real-world clinical application this means that every implant would need to undergo tedious model training sessions, which could be a burden to both patients and medical staff and may hinder a broad clinical adoption. To address this critical limitation, we explored three computational approaches that do not depend on patient individual training while accounting for individual differences in ECoG strip location. In a first approach, the data were spatially extrapolated to a manually defined 38-point cortical surface grid, similar to a previous study^16^ (Fig. 2h). The disadvantage of this however, is a) that extrapolation leads to inaccuracy in spatial estimation and b) large amounts of data would be required to train a grid of the whole brain, which limits the application to cohorts with very similar electrode locations.

To overcome these shortcomings, we have developed a connectomic approach for across-patient decoding that optimally accounts for the specific recording localization while being generalizable across the whole brain. It builds on functional or structural connectivity fingerprints extracted from brain signal recording locations in normative space. In brief, voxel-wise correlations between decoding performance and whole-brain connectivity maps seeded from channel MNI coordinates were calculated to identify an optimal connectomic template fingerprint for movement decoding (so called connectomic decoding network map) across all subjects (Fig. 2i). This allows for an optimized a priori channel selection in real-time, by identifying the individual recording channel that has most network overlap with the optimal template. Finally, we have transformed neural features from the selected channel into a lower dimensional embedding^13^. For this, a five-layer convolutional neural network with a temporal filter length of 1 s was trained using the InfoNCE (Noise-Contrastive Estimation) contrastive loss function^17^. The resulting embeddings showed exceptionally high consistency across subjects as investigated with linear identifiability (Fig. 2j,k).

All three approaches reached significant above chance balanced accuracy and movement detection metrics (Table 1; Fig. 2l,m, all p<0.05) for cross-validation without patient-individual training within and across cohorts, and even leaving entire cohorts out. This indicates high generalizability across movement types, neurological disorders, recording setups and individual implant trajectories. In addition to the conceptual advantage to account for specific recording location and underlying brain network affiliation, the connectomic approach with contrastive learning (CEBRA) significantly outperformed the linear model for the most challenging across cohort cross-validation in sample-wise balanced accuracy (p=0.001) and overall achieved highest average performances (see Table 1).

To investigate the individual variability of trainer vs. learner performance and potentially improve the training process according to diversity related factors, we established a subject-to-subject decoding matrix. This allowed to identify individual subjects that provide outstanding training data for other subjects respectively (Fig. 2n). The average sample-wise decoding performance trained on just the best trainer sub_002 (Berlin) for the remaining cohort of 55 patients was 0.71 ± 0.14 for sample-wise prediction and 0.85 ± 0.2 for movement detection. Conceptually, this process could allow for more fine-grained individualized precision medicine approaches that can account for diversity in all its humans forms, e.g. by matching training data to demographic or genetic profiles of patients receiving brain implants.

Finally, after successfully training across patient models offline, we validated the approach prospectively in a newly recruited patient in Berlin (Supplementary Video 1). Importantly, pretrained patient naïve models outperformed individually trained models within the same recording session, with best performance of the model trained on all patients from the Berlin cohort (sample-wise balanced accuracy: 0.71; movement detection rate: 0.97), with slightly lower performances for a model trained on the patient individual data within the same session (45 movements; ~9 minute recording; sample-wise balanced accuracy: 0.67; movement detection rate: 0.97) (Fig. 2o,p). In a third run, we could further validate the patient to patient decoding results, by demonstrating that a model trained on the single best trainer (sub_002) reached comparable performance to the model trained on the patient individual data.

### Decoding emotions from subgenual cingulate cortex in major depressive disorder

Invasive brain signal decoding has clinical utility beyond movement decoding. In the following we highlight the clinical potential of *py_neuromodulation* for brain circuit discovery in the neuropsychiatric domain. In the future, closed-loop therapies for affective disorders may adapt neuromodulation to concurrent mood or may support patients in the valuation of perceived emotion^18,19^. Here, we employed *py_neuromodulation* to investigate optimal circuits, target features and computational approaches to decode perceived emotion from the primary DBS target for major depressive disorder, the subgenual cingulate cortex (SCC).

**Figure 3:**
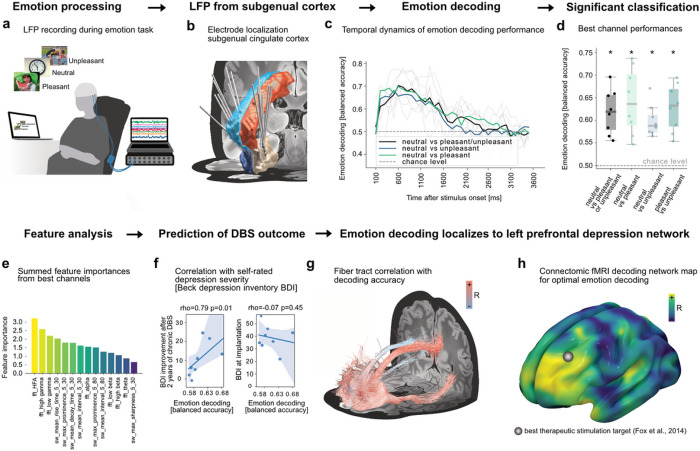
Emotion decoding using LFP signals from subgenual cingulate cortex in patients with treatment-resistant depression. Eight subjects undergoing DBS surgery performed an emotion task (a) with visual stimuli of negative, neutral, and positive valence. Electrode locations are visualized alongside the anterior- (red and light blue) and subcallosal cingulate cortex (white and dark blue, Harvard-Oxford atlas^28^). Balanced decoding accuracies (c) rose from 150 ms after onset, peaked at 600 ms and decayed until 1600 ms post stimulus. Best channel performances showed above chance emotion decoding across subjects (all p<0.05). (e) Best performance channels revealed highest feature importances for FFT gamma features followed by different temporal waveform shape features. (f) Best performances correlated with DBS induced Beck Depression Inventory (BDI) changes 24 months following DBS implantation (rho=0.79, p=0.01). Performances were, however, not significantly correlated to baseline BDI scores. (g) Significant fiber-tracks, FDR (False Discovery Rate) corrected with , predicting emotional state decoding performances showed a clear relation to the left prefrontal cortex. This is reflected in functional and structural connectivity for all patient channels (g) and particularly visible for fiber filtering (h) and fMRI maps (i). The estimated best therapeutic stimulation target from Fox et al 2014 is additionally displayed^25^. All three connectivity models (fMRI, dMRI, fiber filtering) could cross-predict left out channel decoding performances (Supplementary Fig. 1,2).

Machine learning decoders were trained on local field potential signals from the DBS electrodes in SCC in eight patients undergoing DBS for treatment resistant major depressive disorder as part of a clinical trial (mean age: 48 ± 11.4, 4 females; Supplementary Table 4). Neurophysiological recordings were conducted extraoperatively while DBS leads were externalized and acquired while patients participated in a visual emotion task (Fig. 3a). Visual stimuli included pleasant, unpleasant, and neutral stimuli from the international affective picture system (IAPS) database (for further information see^20^) and were presented for a duration of 1 s with an inter-stimulus interval of 6–8 s. We used *py_neuromodulation* to estimate a unique and novel feature set that included temporal waveform features, such as discharge prominence, sharpness, decay and rise time, and peak and trough interval in addition to traditional oscillatory FFT features.

Logistic regression classifiers were trained to distinguish emotional valences using samples during stimulus presentation and cross-validated within patients as described above (Fig. 3b). Above-chance performance was obtained in all patients, with performance rising at 150 ms and peaking at 600 ms post-stimulus onset (Fig. 3c). Sample-wise performances were above chance-level for all classifications (balanced accuracy 0.62 ± 0.05; neutral vs pleasant: 0.64 ± 0.07, neutral vs unpleasant performances: 0.6 ± 0.03, pleasant vs unpleasant: 0.63 ± 0.01) (Fig. 3d). Decoding performance was driven by oscillations including high-frequency (200–400 Hz) activity (HFA), low- (60–90 Hz) and high-gamma (90–200 Hz) activity, followed by waveform shape features including rise time and prominence (Fig. 3e).

To investigate a potential relationship with clinical scores, we correlated decoding performances from the most predictive channel contrasting neutral vs. positive/negative per patient with Beck’s Depression Inventory (BDI) at time of recording and after six months of chronic DBS. Decoding performance correlated with DBS induced improvement in BDI scores (rho=0.79, p=0.01), but not with concurrent symptom severity (Fig. 3f). The correlation could be driven by optimal targeting rather than by depressive symptoms themselves, which inspired us to explore the underlying whole-brain networks. To this end, we used both dMRI and fMRI connectomics as above and an additional fiber filtering approach recently introduced in the context of DBS for OCD^21,22^. In all cases connectivity fingerprints seeded from LFP channel locations were correlated with channel specific decoder test-set performance. The identified fiber tracts and whole-brain dMRI fingerprints were predictive of decoding performance and robust to leave-one-channel-out (Supplementary Fig. 1; fiber filtering rho=0.48, p<10^−5^, whole-brain dMRI fingerprint: rho=0.38, p=0.002) and leave-one-subject-out cross-validation (fiber filtering rho=0.46, p<10^−5,^ whole-brain dMRI fingerprint: rho=0.46, p<10^−5^). Functional connectivity was robust to leave-one-channel out (rho=0.39, p=0.001) but not leave-one-subject out validation (p>0.05).

A consistent left lateralized prefrontal network emerged across modalities that has direct overlap with network targets for the treatment of depression for transcranial magnetic stimulation and has relevant similarity with networks associated with mood change^23^, alleviation of depression and affective changes with subthalamic DBS in Parkinson’s disease^24,25^ (Fig. 3g,h, Supplementary Fig. 2). In the future, emotion decoding may become relevant to adapt neurostimulation control and select optimal targets from electrophysiological biomarkers and connectomics^5,26,27^.

### Optimization of seizure detection parameters for responsive neuromodulation in epilepsy patients

Responsive neurostimulation (RNS; Neuropace) is a closed-loop stimulation device for treatment for medication resistant epilepsy^29^. The device is comprised of a cranially mounted battery and processor connected to two electrodes which can be either an ECoG strip or a stereoencephalography (sEEG) depth-electrode in proximity to the putative seizure focus. RNS promises a superior reduction in the number of disabling seizures by processing LFP signals using programmable detectors of seizures (i.e. ictal) states to deliver temporally targeted stimulation. However, mechanisms of closed-loop stimulation for epilepsy are not well understood. For some patients, this therapy can be life-changing with complete remission of seizures, while for others the therapeutic success remains below expectations^2^. A key differentiating feature versus open loop therapies is the ability to target stimulation to specific neurophysiologic states (such as ictal, inter-ictal, or quiescent) via physician-selected detection parameter settings. Divining appropriate settings from this vast parameter space is a largely manual process taking months or years, if ever, to achieve optimal sensitivity and specificity^30,31^. Improvements in parameter selection may lead to faster and better clinical outcomes and identify new features of interest.

**Figure 4:**
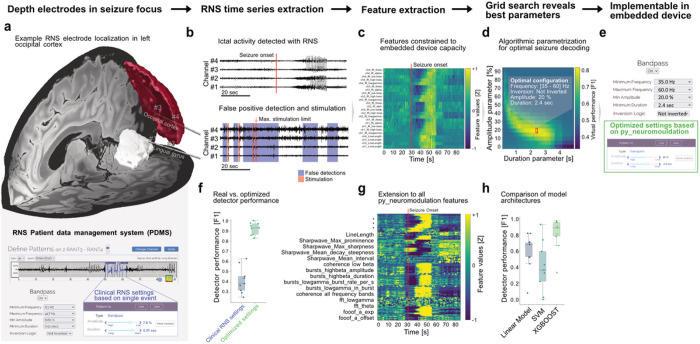
Predictive parameter identification for seizure detection in responsive neurostimulation (RNS). (a) RNS data was either acquired from two lead depth electrodes (eight patients) or a cortical electrode in addition to a depth electrode (one patient). Two lead depth electrodes are visualized using Lead-DBS^36^ for an exemplar patient. The Harvard-Oxford atlas^28^ parcellation shows the lingual gyrus (white) and the left lateral occipital cortex superior division (red) that are penetrated by the electrodes. (b) In an exemplary recording, from a baseline programming epoch without stimulation artifacts, ictal onset characteristics across the four recording channels are displayed (top). RNS detectors can produce false positive seizure detections even in the absence of seizure activity (bottom) that can occur so frequently that no further therapy is provided (maximum stimulation number reached), even though no ictal activity is present (as defined by the epileptologist annotation). (c) FFT and line-length features were computed similar to the embedded RNS algorithm with *py_neuromodulation*. The exemplary ictal recording shows clear seizure induced changes. (d) By combining these features with expert seizure annotations, optimal detection parameters were extracted in a grid-search to optimize the F1 score for seizure detection. (e) The RNS patient data management system (PDMS) provides the “SimpleStart” algorithm, in which detection programming settings are automatically inferred based on a single ictal event. Instead, we propose using optimized parameters based on machine learning models that are trained on expert annotations across hundreds of events with *py_neuromodulation*. This may hold the potential for improved true negative predictions and increased F1 scores (f). It can become even more promising when more complex feature sets and machine learning algorithms will be implemented in the brain implant. (g) We show that the extension to multiple additional features (e.g. bursting, fooof, temporal waveform shape, etc.) can further increase performance with robust three-fold cross-validation. (h) Gradient-boosted decision trees using the XGBOOST framework were outperforming linear models and support vector machines for seizure prediction using all computed features and may provide an optimal trade-off between complexity and interpretability in future neurotechnology.

Here, we aim to inspire new ways to improve seizure detection accuracy by constraining the decoding platforms to the specifications of clinical brain implants and suggesting improved parameters from offline predictions that are implementable and testable through the clinical patient data management systems (PDMS) provided by Neuropace. For this, we analyzed over 100 hours of invasive human brain signals from neocortical depth electrodes (see Fig. 4a for an example) recorded with RNS devices in a cohort of nine epilepsy patients (mean age: 35.3 ± 8.2, 8 females, all focal epilepsy; mean number of available recordings: 636.9 ± 366.4, mean recording duration: 71.07 ± 10.78 s, Supplementary Table 5). With the aim to reduce the false positive rate of the implemented seizure detector, we performed a systematic feature optimization in a simulation of the RNS bandpass detector algorithm. This embedded algorithm is normally programmed by the clinical team via the selection of a single ictal event (“SimpleStart”) which provides the foundation for a semi-automatized parametrization of detection settings for this specified event in the programming environment.

We extracted brain signals and detection and stimulation parameters from RNS implants (Fig. 4b) using a previously described database access pipeline^32^. Individual recordings were annotated by a certified epileptologist for electrographic presence and onset of seizure activity. The resulting annotations served as ground truth for using machine learning methods that can identify embedded bandpass algorithm parameters using our platform. Three parameters need to be defined for the detector by the clinical team: i) the threshold direction, i.e. whether increase vs. decrease in band power is associated with ictal activity, ii) the corresponding threshold amplitude and iii) the required duration the threshold is crossed for a seizure event to be detected. We optimized these parameters for sample-wise seizure classification to maximize the F1-score. We focused on F1 scores instead of balanced accuracy, because ‘seizure present’ (true positive) predictions are more critical for clinical scenarios than correct ‘seizure absent’ (true negative) predictions, which is why they are commonly used as a metric for RNS seizure prediction performance^33^.

Brain signals aligned to seizure onset revealed high-frequency synchronization followed by activity in lower frequency bands, as previously described (Fig. 4c)^34,35^. An exemplar grid-search matrix spanning the threshold direction, amplitude and duration of each feature, demonstrates how *py_neuromodulation* can directly provide access to RNS detector parameters that can be used in the embedded framework and implemented through the patient data management system (Fig. 4d,e). The identified parameter combination significantly reduced false positives, while maintaining stable true positive rates leading to overall performance increases (F1 score for original RNS settings: 0.41 ± 0.12, F1 score for our optimized settings 0.92 ± 0.06) (Fig. 4f). It is important to note that the embedded RNS programming environment does not have cross-validation implemented which was mirrored in our simulation, and that RNS data are in part recorded because of true or false- positive seizure detection (114.16 ± 86.42 minutes out of 6515 minutes of total recording time were classified as ictal by expert annotations). Thus, the presented data should rather be interpreted as a proof-of-concept that requires further validation in prospective clinical trials.

To highlight the utility of multivariate brain signal decoding for seizure detection beyond the RNS device limitations we further evaluated an extensive set of 264 features (66 features per channel) (Fig. 4g). We then assessed the performance of linear models, support vector machines (SVM), and gradient boosted decision trees (XGBOOST) using a 3-fold cross-validation as before. XGBOOST achieved best performances (F1 score: 0.8 ± 0.2) and outperformed linear models (Linear Model 0.56 ± 0.23, Support Vector Machine 0.4 ± 0.3) (Fig. 4h), potentially by capturing non-linear interactions more robustly than SVMs and linear models.

Our results emphasize two key aspects regarding the utility of our platform for the development of decoding algorithms for brain implants: i) it can be of direct use for the parametrization of currently available and embedded algorithms in available brain implants, ii) it enables the discovery of optimized feature sets and machine learning methods for the next generation of clinical brain computer interfaces for the treatment of epilepsy and other brain disorders.

## Discussion

We introduce *py_neuromodulation*, a modularized invasive brain signal decoding platform for real-time implementation in the context of clinical brain computer interfaces. We highlight three major advances of this platform in use cases covering key innovation domains for neurotechnology across more than 123 hours of brain signals from seventy patients who have undergone treatment with brain implants.

First, we demonstrated the ability to decode behavior across disease entities, movement types, acquisition systems and cohorts from the US, Germany and China. Importantly, our pipeline achieved high decoding performance in a prospective patient naïve real-time validation of pretrained movement decoding models. We propose a combination of connectomics based channel selection and contrastive learning (CEBRA) for across patient decoding that circumvents tedious patient individual training that would obviate widespread clinical adoption of brain signal solutions. Moreover, we show that training data from an individual subject can generalize to other subjects, which holds promise for across-patient decoding applications even in rare diseases. It may further allow to account for different dimensions of human diversity, such as anatomical variation, genetic differences and/or disease severity.

Next, we highlight the utility of our approach beyond movement disorders, by decoding perceived emotional valence in patients with treatment resistant depression. Here, we additionally demonstrated the predictive relevance of feature sets beyond oscillatory activity such as waveform shape for emotion decoding. Combining brain signal decoding with connectomics in this use case revealed the whole-brain circuits underlying emotion decoding in a left prefrontal network. The resulting network showed resemblance to previously published and validated neuromodulation targets and lesion networks that were reported to affect mood^23,24,37^ and may indicate optimal treatment targets to refine DBS for depression.

Finally, we showcase opportunities to directly improve parametrization of RNS detectors for specific neurophysiologic states of seizure networks, such as detection of ictal states, through feature identification using available ECoG recordings and simulation of available detector settings for the treatment of epilepsy. The vast parameter space of closed-loop systems, such as RNS, present an optimization challenge significantly better-suited to machine learning models than the current length and error-prone manual process. Further, the features of seizure networks may change over time, further delaying optimization as physicians struggle to keep up with a moving target. Timely implementation of optimal seizure detection settings, even as the network features evolve, is imperative to directly improving clinical outcomes. Prospective validation of this approach in clinical trials may lead to lower false positive stimulation rates, higher temporal specificity and lower stimulation dose, and promise both faster and superior clinical outcomes with fewer side-effects.

Our use-cases are built on the prediction of brain states in data from neurosurgical patients who have undergone treatment with neural implants for brain stimulation. However, an important limitation is that through the retrospective nature of these use-cases, we did not evaluate the efficacy or temporal precision of resulting brain stimulation algorithms. Nevertheless, decoding performances were investigated in the presence of clinical brain stimulation in two out of three use cases, namely STN-DBS for PD and responsive neurostimulation for epilepsy. Moreover, its application is particularly promising for the combination of neural population activity and LFP for the prediction of low-dimensional decoding targets. Thus, it may have additional utility but will not replace the complex algorithm development in other research domains, e.g. using single unit activity in the context of spatial navigation^38,39^ or for complex neuroprostheses to recover speech or to provide brain to text communication^40,41^ that have reached astonishing performance through extensive optimization of recording techniques, model architectures and control policies tailored to these specific applications. Finally, some similar and many other use cases for machine learning based invasive brain signal decoding have been reported before^1,4,5,16,27, 42–45^.

What makes *py_neuromodulation* in this context unique is that it provides a collaborative open-source platform for standardized and reproducible translation of offline decoding into clinical applications. Meanwhile, it extends previous methods for feature extraction and combines them with novel solutions to across patient decoding with MRI connectomics. With this, it is prepared to serve large-scale multicenter collaborations as demonstrated with this study, to develop machine learning models that can generalize across centers and patients to inspire the next generation of closed-loop neurostimulation.

In the future, our platform may provide the foundation for network specific and brain-state dependent closed-loop neurostimulation approaches that dissociate symptoms^46^, side-effects^1^ and volitional behavior^4,43^ to offer the best therapy for the individual situation our patients are facing. To give a practical example, symptom specific brain networks have recently been discovered for STN-DBS in PD that differentially underlie improvement in tremor, bradykinesia, rigidity and gait disturbance^47^. The network description however, is static in nature, while symptoms wax and wane. Brain signal decoding may help these networks to come to life in a dynamic closed-loop neurostimulation approach by informing both decoding and stimulation models of symptom specific circuits to optimize symptom decoding and adaptation of stimulation right at the time they occur.

In conclusion, *py_neuromodulation* provides a novel open-source platform that has the potential to democratize and standardize brain signal decoding in the development of next generation neurotechnology for closed-loop neurostimulation with clinical brain computer interfaces.

## Methods

The open-source brain signal decoding platform *py_neuromodulation* and its’ algorithms were developed in the Python programming language. All code and documentation is openly accessible as a Python package (licensed under an open-source-compliant MIT license). The module supports Python version 3.10 or later and is maintained on GitHub (https://github.com/neuromodulation/py_neuromodulation; see the Code availability statement). The GitHub repository includes instructions for installing and contributing to the package, and the documentation materials that are further hosted on (https://neuromodulation.github.io/py_neuromodulation/). The platform handles real-time data streams from neural processors equally to offline data files in Brain Imaging Data Structure format (BIDS)^48^, i.e. data can be streamed from the API of a neural processor or from an offline stream that is simulated from datafiles. This secures equal performance of brain signal decoders in offline and real-time situations, readymade for direct clinical adoption. Preprocessing, re-referencing, normalization and extraction of eight FFT multiband features for state predictions from a single channel on contemporary hardware (Intel i7 laptop computer) takes less than 10 ms.

### py_neuromodulation parametrization

#### Initialization of data streams

*py_neuromodulation* requires the initialization of an offline or online stream object with the sampling frequency of the recorded data and two parametrization files: *nm_channels* and *nm_settings* (see for parametrization of available features Supplementary Table 1, preprocessing Supplementary Table 6 and postprocessing Supplementary Table 7). The *nm_channels* file provides channel specific information such as re-referencing, decoding target selection, and channel renaming (Supplementary Table 8). It builds upon the *channels.tsv* file of the BIDS specification for intracranial electroencephalography^48^ and adopts parameters defined therein such as *channel name*, type and *status*. The *nm_settings* file specifies parameters for feature estimation and pre- and postprocessing. Both files can either be created during runtime or be loaded from disk by providing paths to a *nm_channels*.*csv* and *nm_settings*.*json* file. After initialization, all settings are tested, and the stream object’s run function can be called for batch-wise feature calculation. For offline applications, the stream generates sequential data batches that are processed to simulate a real-time setting. The size of the data batch is specified in *nm_settings*. In the simplest case, the stream can then be given the entire array of recorded data. The platform additionally provides an interface to automatically load data and metadata such as sampling frequency, channel names and types, and electrode locations from files in BIDS format using *MNE-BIDS*^49^. When data is directly streamed from a neural device, the same procedure can be called to process arriving data batches in precisely the same way as done for offline analysis and training. When the streams’ run function is terminated, all feature and parametrization files are saved for further analysis. Various visualization and machine learning analysis functions can then be utilized through the *nm_analysis* module.

### Feature estimation

A variety of features were implemented that can capture different characteristics of neural time series data from multiple data analysis domains, which may provide complementary information on brain states and behavior^8^. For extraction of oscillatory features (FFT, STFT, Finite Impulse Response (FIR) bandpass filtering) is implemented in *py_neuromodulation* with frequency adapted time windows to optimize information content. Spectral parametrization can separate periodic and aperiodic components of power spectra that can provide differential information on neural processing^7^. We developed a wrapper for the FOOOF-toolbox that allows for computation of aperiodic and periodic parameters for real-time decoding. In the temporal domain, the presence, amplitude and duration of oscillatory bursts were shown to provide complementary information to average oscillatory power^50–53^. For each feature window, mean burst duration, amplitude, burst rate per second and an in-burst state can be estimated for several frequency bands and different thresholds across a variable time window.

In addition to neural oscillations, temporal waveform shape has previously been demonstrated to reflect different pathological and physiological states^6,54^. For multielectrode recordings, interregional functional oscillatory connectivity can provide important information for decoding applications^55^. Therefore, a real-time compatible coherence computation across channels and frequency bands was implemented, as well as a wrapper for the MNE-Connectivity toolbox (https://mne.tools/mne-connectivity/stable/index.html).

Temporal waveform shape estimation is implemented in *py_neuromodulation* in a batch-wise manner for signal troughs and peaks for which the *prominence* measures the mean amplitude difference of the trough and the surrounding peaks (or peak with surrounding troughs):

$$V_{prominence}=\left|\frac{V_{peak-left}\;+\;V_{peak-right\;}}{2}\right|-V_{trough}$$

The *sharpness* measures the voltage deflection of each trough/peak with respect to the voltage amplitude at time points preceding and following 5 ms:

$$V_{sharpness}=\frac{\left(V_{trough}-V_{trough-5ms}\right)\;+\;\left(V_{trough}-V_{trough+5ms}\right)}{2}$$

Additionally, the interval between troughs/peaks can be calculated, as well as the width of the surrounding peaks/troughs, and the rise and decay time and steepness. The maxima of resulting measures per time window can then be used for model training and decoding. Nonlinear measures for dynamical systems (*nolds*) estimate trends in the data and characterize different metrics of the time series dynamics. The features are obtained through a wrapper for the *nolds* toolbox, and can calculate sample entropy, correlation dimension, Lyapunov exponent, or detrended fluctuation analysis of raw or bandpass filtered data^56^. Additionally, Hjorth features can be calculated for raw data or bandpass-filtered data separately for different frequency bands^57^. Kalman filters can be used for post-processing of oscillatory features to reduce noise^45,58,59^.

### Feature analysis and decoding

In the *nm_analysis* module, features can be visualized across time for specific recording time windows or averaged for a target signal condition (Fig. 1c). Electrode locations can be plotted on a cortical surface and color coded with obtained features or performances. Different decoding methods were implemented in the *nm_decode* module. Machine learning methods can be evaluated for different validation strategies for individual or combined channels, e.g. as supported by scikit-learn^9^. Multiple samples can be combined for feature analysis for e.g. Wiener filter methods^45^. Principle component analysis or Canonical correlation analysis can be used for dimensionality reduction. Features can, in addition, be selected through the Maximum Relevance and Minimum Redundancy method (https://github.com/smazzanti/mrmr)^60^. Unbalanced datasets can be resampled through random over-or undersampling using the imbalanced-learn framework^61^. Since many decoding applications require performance evaluation on not only a sample, but a consecutive group-wise level, detection accuracies can be calculated for a minimum modifiable number of high consecutive predictions, as implemented for movement detection (Fig. 2).

### Decoding without patient individual training

Decoding from brain implants without patient individual training needs to account for large individual variance of electrode locations and anatomy^16^. We implemented three methods that can achieve this: grid point interpolation, spatial correlation of whole-brain network for channel selection and contrastive representation learning using CEBRA. Grid-point decoding relies on a predefined cortical or subcortical grid definition in a common space (e.g. the non-linear asymmetric version of the Montreal Neurological Institute space MNI152NLin2009bAsym). Data from individual electrode contacts $x_{m}$ are interpolated to common grid points $x_{\text{gridpointn}}$, and weighted by their normed distances dist⁡(n,m) to each grid point:

$$x_{gridpointn}=\frac{1}{\sum_{contactm=1}^{M}\;\;dist(n,\;m{)}^{-1}}\sum_{contactm=1}^{M}\;x_{m}dist(n,m{)}^{-1}$$

All electrode contacts M are selected through a maximum distance threshold between each electrode contact m and the respective grid point n. In this way, localized information is grouped to grid points that are shared across patients. Decoding without patient individual training can then be performed using the grid point data. Therefore, this approach identifies a projection to a standardized space that is comparable to EEG layouts.

Alternatively, single ECoG or depth electrode channels can be selected through optimal connectivity. Functional and structural connectivity measures can for example be calculated within the Lead-Mapper tool of the Lead-DBS toolbox^36^. An optimal connectomic decoding network map is then constructed through voxel-wise correlation of connectivity profiles with decoding performances from offline training^11^. This allows prospective channel selection even in previously unseen subjects by comparing the network fingerprints of all available channels to identify the one that is most similar to the optimal connectomic decoding network map that was informed by other subjects.

The selected channels can then be used for learning an embedding based on the *py_neuromodulation* feature space using CEBRA^13^. Contrastive learning can thus be used to train a neural network to minimize distances between behaviorally and temporally close samples and maximize distances from temporally distinct samples. The obtained embeddings can be compared by computing a linear mapping from one embedding to another, resulting in a “consistency map”^62^. A binned histogram can be computed to take target information into consideration and correlate the resulting embeddings. This provides a powerful ground for across-patient predictions for human brain implants.

### Use-case specific patient details and decoding methods Movement decoding from ECoG signals in epilepsy and PD patients undergoing STN-DBS

Invasive brain signals were analyzed from 56 subjects across four cohorts. The data acquisition methods varied across the four movement decoding cohorts. Patients with Parkinson’s disease (Berlin, Beijing, Pittsburgh) underwent additional ECoG recordings in the context of neurosurgery for subthalamic DBS implantation. Motor sign severity was assessed preoperatively using Unified Parkinson’s disease rating scales (UPDRS-III total score) for all patients. Berlin subjects (12 PD patients, 5 females) were recorded postoperatively through externalized six or twelve contact ECoG electrodes (Ad-Tech 1×6 layout, contact area: 12.56 mm^2^; Ad-Tech 1×6 layout, double sided, contact area: 12.56 mm^2^) using a Neuro Omega (Alpha Omega) acquisition system (Berlin subjects 1–2) or with the SAGA amplifier (TMSi; Berlin subjects 3 to 12). In total, there were 38 recordings, of which 17 were performed ON dopaminergic medication (11 patients). All patients were instructed to perform voluntary hand movements with the upper limb contralateral to the hemisphere of ECoG electrode placement. In a sub-cohort of six patients bilateral monopolar STN-DBS was delivered with clinically effective stimulation settings, as determined by monopolar review (stimulation frequency: 130 Hz; pulse width: 60 μs; mean amplitude: 2 mA). Data from the Beijing cohort was also recorded in PD patients undergoing STN-DBS using externalized ECoG electrodes (n = 10, 8 females) using a JE-212 amplifier (Nihon Kohden). Patients carried out a voluntary button press task. Six subjects were measured in the medication ON condition (6 recordings). Data in the Pittsburgh cohort (n = 15 patients, 3 females, 73 recordings) was recorded intraoperatively during STN-DBS implantation using a Grapevine neural interface processor (Ripple). All patients were in medication OFF condition and performed a cued Go/No-Go task that was previously described^63^. The electrode setup varied across Pittsburgh patients: Ad-Tech electrodes using 6 (layout: 1×6; contact area: 12.56 mm^2^), 8 (layout: 1×8, contact area: 12.56 mm^2^), or 28 contacts (layout: 2×16, contact area: 3.14 mm^2^) were used. All data from epilepsy subjects were measured in the Epilepsy Monitoring Unit (EMU) (n = 18, 7 females) using Synamps2 amplifiers (Compumedics Neuroscan) at the University of Washington and Washington University in St. Louis. The data was previously made publicly available in the publication “A library of human electrocorticographic data and analyses”^14^ and was also analyzed in a former study^64^. Here we used the “motor_basic” dataset including hand movements with “synchronous flexion and extension of all fingers”.

For prospective model validation and to showcase the real-time applicability of the *py_neuromodulation* platform, we recruited an additional PD patient at the Movement Disorder and Neuromodulation Unit at Charité Universitätsmedizin Berlin (age: 65 years, female). ECoG recordings were measured from an Ad-Tech electrode (1×6 contact layout, contact area: 12.56 mm^2^) and a SAGA (TMSi) amplifier. The patient performed voluntary wrist rotations as described for the Berlin cohort above.

To demonstrate movement decoding across centers, movement types, and diseases, we computed features and applied standardized machine learning decoding methods across the four cohorts described above. Data from all subjects was streamed in batches and the following processing chain was applied to every batch: All data were resampled to 1 kHz, re-referenced by subtracting the common average, and FFT features were calculated for different frequency bands: θ (4–8 Hz), α (8–12 Hz), low β (13–20 Hz), high β (20–35 Hz), low γ (60–80 Hz), high γ (90–200 Hz), and high-frequency activity (200–400 Hz) at a temporal resolution of 100 ms. All features were z-score normalized across the past 30 s and clipped at minus and plus three, to normalize the feature range across subjects and cohorts. Ridge-regularized logistic regression models were trained based on motor output that was provided as the target channel using balanced class-weights for prediction of movement presence using scikit-learn^9^. Performance on each channel was assessed using the balanced accuracy metric, which considers imbalances in target distributions. Three-fold cross-validation was employed on consecutive data segments without shuffling individual samples, which can lead to data leakage through temporal correlation of feature estimates.

For across-patient decoding, electrode contact localization was performed or available from neuroimaging as a prerequisite to the three different approaches described above: first, the features were projected to a custom 38-point sensorimotor grid in MNI space with a maximum channel to grid-point distance of 20 mm. Logistic regression classifiers were trained on grid points instead of channels for each patient. This circumvents the requirement to select single channels at the patient level. Alternatively, in a second approach, channel selection was performed using connectomics. A spherical seed (4 mm radius) was used as a region of interest to compute the connectivity profiles for each recording contact using Lead-Connectome Mapper in the Lead-DBS toolbox^36^. An optimal connectomic network map for movement decoding was calculated using voxel-wise correlations of channel-specific balanced accuracies connectivity profiles. *py_neuromodulation* was then used to validate the computed connectomic decoding network map across different cross-validation strategies: leave one subject out within cohorts, leave one subject out across cohorts, and leave one cohort out cross-validation. CEBRA contrastive learning was used with the “offset10-model” five-layer convolutional neural network (32 hidden units for the first layer, followed by three convolutional *skip layers* with each 32 hidden units and a four-dimensional convolutional output layer). The *skip layers* resembled a bottleneck across the temporal filter dimension from 10 to 3 samples. A Gaussian Error Linear Unit (GELU) activation function was used for each layer, and normalization was applied to the output layer. The “auto” temperature mode was specified with a learning rate of 0.005. 1000 training iterations were used with a batch size of 100. Positive sampling with the InfoNCE loss function was performed by sampling time samples within a “time_offset” of one and samples respective to the auxiliary movement variable as defined in the target channel. To approximate the movement kinematics, a gaussian filter with sigma 1.5 was used to smooth each label, and color-code the exemplar embeddings. All code required for reproduction of the movement decoding analysis was made publicly available in a dedicated GitHub repository (https://github.com/timonmerk/AcrossCohortMovementDecoding).

All preprocessing, feature estimation, and postprocessing steps of *py_neuromodulation* were optimized for real-time application. Raw data visualization in real-time is realized using BrainStreamingLayer (https://github.com/bsl-tools/bsl), and features as well as model predictions are visualized using Timeflux^65^. All features are saved using Timeflux in the hdf5 format for post-hoc training and model comparison. The *nm_settings* and *nm_channels* specification files can be identical for offline and online analysis. To demonstrate its utility in the movement decoding scenario for a newly recruited patient, the offline data batch generator was replaced by the API of a hardware acquisition system (TMSi SAGA) to stream signal batches directly from the neural implant in real-time. In an initial movement recording run (as described for the remaining Berlin cohort), a pretrained model from all other Berlin cohort patients was applied and real-time decoding performance was objectified for the patient naïve “plug & play” application. Simultaneously, data was stored, and used for offline training of a patient individual model after the first run was concluded. Performance of this patient individual model was validated in real-time in the subsequent run. In a third run a pretrained model was tested, which was based on the single subject with best training outcome from the Berlin cohort (sub_002) to prove that patient to patient prediction is viable at the single subject level, even in prospective real-time applications. All code required for reproduction of the real-time implementation using the TMSi SAGA acquisition system was made publicly available in a GitHub branch of the *py_neuromodulation* repository (https://github.com/neuromodulation/py_neuromodulation/tree/realtime_decoding_analog_tr).

### Emotion decoding in patients with treatment resistant depression undergoing SCC-DBS

Data from treatment-resistant depression subjects (n = 8, 3 females) engaging in an emotion picture viewing task was retrospectively analyzed^20^. Patients were resistant to treatment of cognitive behavioral therapy, and or electroconvulsive therapy and underwent a clinical trial at Charité – Universitätsmedizin Berlin. Supplementary Table 4 displays the following details: age, preoperative and 24 months DBS BDI follow up scores as reported in^66^. Data was recorded using DBS electrodes (Medtronic model 3387). Through post-operative fMRI images, electrode locations were estimated through the Lead-DBS software and coregistered to MNI space^36^. Patients were participating in a passive picture viewing task showing pleasant, unpleasant and neutral stimuli of the International Affective Picture System (IAPS)^67^. Pictures were presented with a duration of 1 s and with a randomized inter-stimulus interval of 6–8 s. Additionally, pictures were matched for mean neutral valence and arousal. All recordings were referenced bipolarly and band-pass filtered between 0.5 and 250 Hz through the recording hardware (D360 Digitimer Ltd).

Data with epochs containing different emotional stimuli were concatenated to build the simulated data stream of invasive recordings from patients with treatment-resistant depression. The time periods containing stimulus presentation were labeled according to the emotion class. Since data was already hardware bipolar re-referenced, no rereferencing was specified. Raw data batches from the signal stream were notch-filtered, and FFT features (θ (4–8 Hz), α (8–12 Hz), low β (13–20 Hz), high β (20–35 Hz), low γ (60–80 Hz), high γ (90–200 Hz), and high-frequency activity (200–400 Hz)), as well as temporal waveform shape features (max prominence, mean interval, max sharpness, mean decay time, mean rise time) were calculated. Performances for different classification problems were obtained using a ridge-regularized and class-weight balanced logistic regression model as above.

Healthy subject functional and diffusion-weighted structural MRI connectivity profiles, as well as structural connectivity using fiber-tracts, were calculated for each recording contact location (region of interest seed with 4 mm radius) using openly accessible connectomes (Yeo et al 2011^68^ for fMRI (n = 1000) and Human Connectome Project data^69^ for dMRI and fiber-tracking (n = 985)). This allowed to calculate optimal connectomic emotion decoding network maps that were validated in leave one channel and leave one subject out cross validation approaches (Extended Data Fig. 1). The Lead Connectome Mapper and fiber-filtering was performed using the Lead-DBS toolbox^36,69^. Leave one channel and leave one subject out cross-validation approaches were implemented in *py_neuromodulation*. All code required for reproduction of the emotion decoding analysis was made publicly available in a GitHub repository (https://github.com/timonmerk/TRD_pynm).

### Seizure decoding in epilepsy patients with responsive neurostimulation implants

Intracranial data from 9 patients was obtained through the Biophysically Rational Analysis for Informed Neural Stimulation (BRAINStim) platform developed from the Brain Modulation Lab^32^. Patient individual information such as number of lead depths and ECoG strip electrodes, recording locations, time since RNS implantation, and seizure reduction scores are displayed in Supplementary Table 5. The RNS device can be programmed for storage of scheduled recordings, as well as high risk seizure recordings (called Long Episodes). The concatenated recordings were further annotated for seizure presence and aligned with detection and stimulation events that the BRAINStim platform further logs from the Patient Data Management System (PDMS).

### Offline parametrization for seizure presence decoding

The previously published BRAINStim pipeline allows to access streamed recordings from the responsive neurostimulation (RNS) implant^32^. Electrophysiological data from either lead-depth or ECoG strip electrodes was stored across multiple months in addition to stimulation and detection events in a database. Each recording was linked to a set of stimulation and detection events, as well as seizure presence annotations from an expert neurologist (V.K.). *py_neuromodulation* was used to first assess the performance of different detection settings using the bandpass detector. FFT features were calculated for different frequency bands (θ (4–8 Hz), α (8–12 Hz), low β (13–20 Hz), high β (20–35 Hz), low γ (35–60 Hz), broadband (20–120 Hz)) with a feature sampling rate of 1 Hz and segment length of 1000 ms. Feature normalization was omitted to ensure feature discriminability across ictal and non-ictal recordings. Since data was hardware bipolar referenced, no additional software re-referencing was performed. Data segments from two artifact components were identified and excluded from further analysis: First, stimulation events result in the RNS stored data as a “flatline” stationary artifact, which does not contain electrophysiological data. Second, suboptimal amplifier gain settings can result in a “clipping” artifact, where all data is clipped to analog-to-digital converter respective maximum bounds. Both artifact types were identified if the voltage derivative did not change in a time range of at least twelve consecutive samples (48 ms). Since stimulation events induce additionally high amplitude fluctuations in the RNS stored recordings, two seconds following each stimulation pulse were annotated to contain artifacts.

A performance of seizure presence decoding was obtained by optimizing different detection parameters. A recording was classified as seizure if for a defined minimum duration the voltage amplitude crossed a certain threshold. In a grid search, the minimum duration was tested across 50 values from 100 to 5000 ms in steps of 100 ms; and the amplitude was sampled from 20 equally spaced values within the minimum and maximum voltage range. Additionally, each feature could be inverted by the RNS implant, resulting in a three-dimensional grid-search across minimum duration, minimum amplitude, and inversion. The obtained seizure classification was measured as a F1-score and compared against the RNS programmed bandpass-detector prediction performance.

In the next step, several additional *py_neuromodulation* features were computed: raw signal line length, temporal waveform shape features for 5–30 Hz and 5–60 Hz filtered data (temporal waveform-shape features: width, interval, rise time, decay time, rise steepness, decay steepness, prominence, interval, sharpness), bursting features for a 75 percent threshold within a duration of 30 s for signals filtered in the low beta, high beta, and high gamma frequency bands (bursting features: duration, amplitude, burst rate per second, in-burst state), spectral aperiodic components (exponent and offset), and coherence in the high beta and low gamma frequency bands between recording contacts of each electrode as well as across electrodes. Using a non-shuffled three-fold cross-validation, ridge-regularized logistic regression models, support vector machines, and gradient boosted decision trees using the XGBOOST framework were tested^70^. In this manner the potential performance gain of additional features in combination with machine learning models was evaluated. All code required for reproduction of the optimal RNS seizure decoding analysis was made publicly available in a GitHub repository(https://github.com/timonmerk/rns_pynm_optimal_detection_params).

## Figures and Tables

**Table 1:** Leave one subject out and leave one cohort out cross-validation results for generalized movement decoding across patients, diseases and movement types. Decoding performance is depicted as balanced accuracy, which accounts for class imbalances. Single sample estimates provide performance metrics at 100 ms precision. Movement detection estimates were defined as 300 ms consecutive classification output with respect to presence and absence of movement. On average, the connectomic approach combined with contrastive learning (CEBRA) provided best cross-validation performances.

	Grid extrapolation		Connectomics with LM		Connectomics with CEBRA	
	Sample	Movement	Sample	Movement	Sample	Movement
Within cohort	0.71 ± 0.08	0.84 ± 0.12	0.72 ± 0.08	0.86 ± 0.10	0.74 ± 0.1	0.86 ± 0.14
Across cohorts	0.64 ± 0.1	0.78 ± 0.2	0.66 ± 0.09	0.78 ± 0.19	0.68 ± 0.09	0.81 ± 0.16
Leave one cohort out	0.54 ± 0.11	0.61 ± 0.22	0.57 ± 0.1	0.68 ± 0.19	0.6 ± 0.11	0.7 ± 0.19
Average	0.63 ± 0.1	0.74 ± 0.18	0.65 ± 0.09	0.77 ± 0.16	0.67 ± 0.1	0.79 ± 0.16

## Data Availability

Data for movement decoding are available for the Pittsburgh (https://doi.org/10.7910/DVN/IO2FLM) and Washington (https://purl.stanford.edu/zk881ps0522) cohorts. Data from Berlin can be made available conditionally to data sharing agreements in accordance with data privacy statements signed by the patients within the legal framework of the General Data Protection Regulation of the European Union. Data from Beijing can be made available upon reasonable request after approval of the local independent review board of Beijing Tiantan Hospital. All trained models are made openly available for direct implementation in clinical research via the GitHub code repositories.

## Footnotes

Code availability All code and documentation for py_neuromodulation is openly accessible as a Python package, licensed under an open-source-compliant MIT license via https://github.com/neuromodulation/py_neuromodulation. Documentation is hosted on https://neuromodulation.github.io/py_neuromodulation/. All code of the real-time implementation using the TMSi SAGA acquisition system was made publicly available in a GitHub branch of the py_neuromodulation repository (https://github.com/neuromodulation/py_neuromodulation/tree/realtime_decoding_analog_tr). All code of the offline movement decoding analysis was made publicly available through https://github.com/timonmerk/AcrossCohortMovementDecoding. All code of the emotion decoding analysis was made publicly available through https://github.com/timonmerk/TRD_pynm. All code of the RNS parameter analysis was made publicly available through https://github.com/timonmerk/rns_pynm_optimal_detection_params.
